# A Comparative Analysis of the Advances in Scar Reduction: Techniques, Technologies, and Efficacy in Plastic Surgery

**DOI:** 10.7759/cureus.66806

**Published:** 2024-08-13

**Authors:** Christopher R Meretsky, Andreas Polychronis, Anthony T Schiuma

**Affiliations:** 1 Surgery, St. George's University School of Medicine, Great River, USA; 2 General Surgery, St. George's University School of Medicine, Great River, USA; 3 Orthopedic Surgery, Holy Cross Hospital, Fort Lauderdale, USA

**Keywords:** platelet-rich plasma scar management, botulinum scar management, stem cell therapy scar management, microneedling in scar management, laser therapy in scar management, silicone gel in scar reduction, surgical revisions in scar management

## Abstract

The study provides a comprehensive analysis of the latest methodologies and treatments aimed at improving scar management. Scar formation results from the replacement of normal skin with fibroblasts, leading to a structured unidirectional collagen bundle, as opposed to the collagen sheet matrix found in healthy skin. This review categorizes scars into hypertrophic scars and keloids, each with distinct pathophysiological characteristics. It highlights the importance of consistent scar assessment using scales such as the Vancouver Scar Scale and the Patient and Observer Scar Assessment Scale, emphasizing the need for standardized evaluation methods. The study systematically reviews various scar management techniques, ranging from traditional surgical methods to innovative treatments. Conventional approaches such as pressure garments and silicone gel sheeting are explored, noting their roles in maintaining hydration and occlusion. The efficacy of intralesional corticosteroid injections and laser therapies is discussed, with particular attention given to their combined use for optimal outcomes. The review also covers advanced techniques such as microneedling, platelet-rich plasma therapy, and stem cell-based treatments, detailing their mechanisms and potential benefits in scar remodelling. Additionally, the study underscores the emerging role of botulinum toxin A in both preventive and corrective scar treatments, offering promising results in reducing movement-induced scar exaggeration. The systematic review includes a thorough examination of existing literature, clinical trials, and meta-analyses to evaluate the effectiveness of these interventions. It concludes by calling for further research to refine these techniques and enhance their application in clinical practice, aiming to achieve better aesthetic and functional outcomes for patients with scars.

## Introduction and background

The replacement of normal skin tissue by fibroblasts, which heals by resolution rather than regeneration, leaves a scar. The size of the initial wound and the amount of time that passes between an injury and full healing both affect how much scarring occurs. Scar development is caused by a number of causes, including infections, the retention of foreign substances, and healing processes that take longer than two to three weeks. In contrast to the collagen sheet matrix seen in normal skin, structured unidirectional collagen bundles are the hallmark of scars. In contrast to normal skin, this structural variation causes the creation of elevated or conspicuous tissue [1]. Clinically significant scars are classified into hypertrophic scars and keloids. Hypertrophic scars and keloids represent two types of excessive scarring, each with distinct pathophysiological mechanisms. Hypertrophic scars remain confined within the original wound borders and are characterized by the activation of myofibroblasts, driven by a coordinated interplay of various cells such as platelets, macrophages, T-lymphocytes, mast cells, Langerhans cells, keratinocytes, and fibroblasts. This leads to excessive production and altered remodeling of the extracellular matrix, with enhanced expression of types I and III collagen and pathological cross-linking. Hemostasis alterations, increased neovascularization, prolonged re-epithelialization, decreased apoptosis, and heightened inflammation are also key features, with an inflammatory profile showing elevated T helper 2 cells and certain interleukins. Conversely, keloids extend beyond the wound borders, influenced by genetic and environmental factors. They involve abnormal fibroblasts and keratinocytes, increased mast cells, and enhanced expression of fibrosis promoters such as HIF-1α, VEGF, and PAI-1. Altered TGF-β signaling, elevated cytokines and growth factors, and immune alterations, including increased androgen receptor expression and sebum secretion, contribute to their development. Neurogenic inflammation, infection, and mechanotransduction further complicate keloid pathogenesis, making their treatment more challenging compared to hypertrophic scars [2].

Effective treatment for cutaneous scars requires a consistent grading system that takes into account patient comfort and acceptability, as well as variables including pigmentation, vascularity, pliability, thickness, height, and depression. Although they are subjective and have limited sensitivity, a number of measures, such as the Vancouver Scar Scale and the Patient and Observer Scar Assessment Scale, offer reliable and consistent data [3-5]. It is imperative that doctors utilize the same scale for all patients during therapy, even in cases when there is not a gold-standard categorization. The International Advisory Panel of Scar Management (IAPSM) advises taking patient concerns, symptom intensity, scar thickness, and size into account and aids in consistent scar assessment and management. The IAPSM classification includes mature scars (light-colored, flat), immature scars (red, pruritic, painful, potential for linear hypertrophic progression), linear hypertrophic scars (rope-like, red, raised, within original injury borders), widespread hypertrophic scars (widespread, red, raised, often from burns), minor keloids (raised, extending <0.5 cm beyond injury, high recurrence), and major keloids (large, raised, extending >0.5 cm beyond injury, pruritic, painful, continuous growth, very high recurrence [6].

Scar management encompasses various conventional treatments, each with its specific mechanisms and outcomes. Massage therapy, despite limited scientific evidence, is commonly used to alleviate scar-related pain and improve mood. Pressure garments, another standard therapy, reduce collagen synthesis through mechanical compression but have low patient adherence due to discomfort. Adhesive tape support and silicone gel sheeting maintain hydration and occlusion, with silicone gel preferred for its ease of use. Intralesional corticosteroid injections, mainly triamcinolone acetonide, effectively treat keloids and hypertrophic scars, though side effects are common. Laser and light-based therapies, especially pulsed dye lasers, improve scar appearance and are often combined with other treatments for enhanced results. Cryotherapy treats small scars effectively, particularly when combined with corticosteroids, but can cause hypopigmentation. Radiotherapy, used primarily for keloids, poses a malignancy risk. Intralesional injections of chemotherapy drugs such as fluorouracil and interferon show promise but have potential side effects. Other treatments such as tranilast and bleomycin offer additional options but require further research. Surgery remains a primary option for disabling scars, often supplemented by other therapies to minimize recurrence [2]. Surgical scar reduction aims to minimize the incorporation and deformation of normal tissue while improving the aesthetic and functional outcomes of scars. Key principles include making incisions perpendicular to the skin surface, except in hair-bearing areas, and ensuring they align with relaxed skin tension lines (RSTLs). Atraumatic tissue handling, judicious undermining, and tension-free wound repair are critical for optimal healing [7]. Microneedling (MN), or percutaneous collagen induction therapy, is widely used in dermatology for skin rejuvenation, tightening, scar remodeling, and hair growth. It is particularly favored for darker skin types (Fitzpatrick IV-VI) due to its low risk of post-inflammatory hyperpigmentation. While popular in Asia and the Middle East, MN has recently gained attention in the US. MN devices, available as rollers, stampers, and pens, can be combined with radiofrequency (RF) to deliver energy below the epidermis, known as fractional RF MN (FRF-MN), minimizing epidermal damage and dyspigmentation [8,9]. Platelet-rich plasma (PRP) is derived by centrifuging a patient's own blood that has four to seven times the baseline concentration of human platelets. It is used in dermatology for anti-inflammatory, wound healing, and cosmetic uses. Growth factors that influence cell proliferation, differentiation, angiogenesis, and chemotaxis are abundant in PRP and include PDGF, TGF-β, EGF, and VEGF. Additionally, it contains bioactive substances that affect inflammation and membrane permeability, such as histamine and serotonin. Pure PRP, leukocyte and PRP (L-PRP), platelet-rich fibrin matrix (PRFM), and leukocyte and PRFM are the four PRP fractions. The most researched type of PRP, pure PRP, is frequently enhanced with platelet activators such as thrombin or calcium chloride [10]. MSC-based therapy has been proven to ameliorate granulation tissue formation, promote re-epithelialization, and decrease side effects such as pain and infection. Despite the promising results, the limited number of clinical trials and the need for long-term follow-up highlight the necessity for further research to fully understand the therapeutic potential and optimize the application of MSCs in burn and scar treatment. Efficient burn wound healing remains challenging, but MSC therapy offers a viable strategy to improve outcomes and reduce scar formation [11]. Botulinum toxin (Botox) has emerged as a promising treatment for scar reduction, offering multiple benefits in both preventive and corrective contexts. By inhibiting scar formation, Botox can prophylactically improve outcomes in excisions where scars may contrast with RSTLs or where hypertrophic scars are to be re-excised. For existing scars, such as traumatic or post-acne scars, Botox helps by reducing movement-induced scar exaggeration without cosmetic drawbacks. Particularly effective on the upper and lower face, Botox is often combined with fillers, resurfacing, and surgical techniques. The intradermal injection of hyaluronic acid (S-HA) and Botox significantly enhances acne scar treatment through dermal expansion and collagen displacement. This method also stimulates neocollagenesis and neoelastinogenesis, lasting over a year. Additionally, "MicroBotox" and "MicroHA" combinations have proven effective in improving acne scars and reducing large pores with minimal side effects. Overall, Botox presents a versatile and effective tool for managing various types of scars [12-14].

## Review

Methods

This systematic review includes articles that were systematically collected by performing Preferred Reporting Items for Systematic Reviews and Meta-Analyses (PRISMA). This study employs a systematic review of existing literature, clinical trials, and meta-analyses to evaluate the scar reduction efficacy of conventional and newer techniques. A thorough literature search was conducted using databases PubMed, Google Scholar, and Cochrane Library. The keywords used include “SURGICAL REVISIONS IN SCARMANAGEMENT”, “SILICONE GEL IN SCAR REDUCTION”, “ LASER THERAPY IN SCAR MANAGEMENT”, “MICRONEEDLING IN SCAR MANAGEMENT”, “STEM CELL THERAPY SCAR MANAGEMENT”, “PLATELET RICH PLASMA SCAR MANAGEMENT”, and “ BOTULINIUM SCAR MANAGEMENT”.

**Figure 1:**
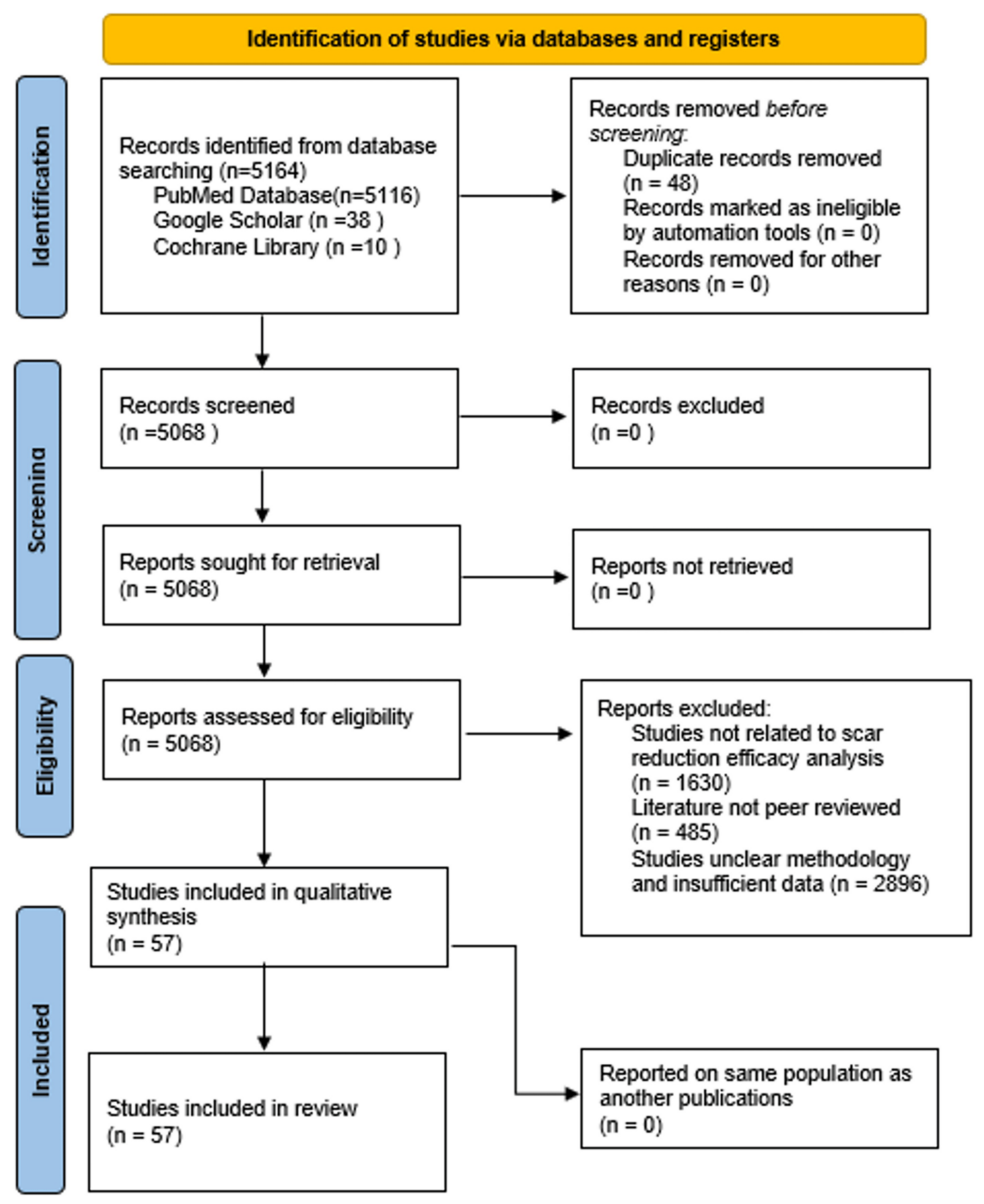
PRISMA flowchart: literature search and study selection n, number; PRISMA, Preferred Reporting Items for Systematic Reviews and Meta-Analyses

The study included peer-reviewed articles, clinical trials, meta-analyses, and systematic reviews focused on scar reduction management with efficacy results published. Articles published between 2000 and 2023 were considered, ensuring a comprehensive understanding of both historical and recent advancements. Exclusion of articles was made for non-peer-reviewed articles, case reports, editorials, studies focusing solely on animal models without human data, and articles not available in English. Figure 1 shows the PRISMA flow chart, which details the systematic process of identifying and selecting studies for the review.

Results

Conventional Methods - Surgical Revisions, Steroidal Injections, and Silicon Gels

Techniques such as layered wound repair and using appropriate suture sizes enhance the strength and appearance of the repair. Common surgical techniques include fusiform elliptical excision, which is ideal for mature, depressed scars aligned with RSTLs, and Z-plasty, which lengthens webbed or contracted scars and realigns them with RSTLs. Multiple Z-plasties distribute tension across smaller segments for inelastic skin and large scars. Variations such as double opposing Z-plasties and four-flap Z-plasties address specific needs, such as limited skin availability or severe contractures. W-plasty and geometric broken line closure (GBLC) irregularize scars to make them less noticeable. V-Y and Y-V advancements lengthen contracted scars and adjust anatomical points. Dermabrasion and microdermabrasion smooth scar surfaces and blend them with surrounding skin, while flaps, grafts, and surgical debulking address large, complex scars. These techniques collectively enhance scar appearance and function while minimizing further tissue disruption [15]. Since hypertrophic scars seldom return after excision, no adjuvant therapy is necessary after the scar is removed. Conversely, keloidal scars may regenerate following excision. This is going to happen in over half of the cases, which means more therapeutic treatments will probably be needed. This needs to be done right away after they are removed. For up to a year, hypertrophic scars typically naturally recede. This implies that waiting until after a one-year period is probably the best time to make surgical adjustments The most widely utilized treatments for managing the recurrence of keloid scars are radiation therapy and corticosteroid injections. Combining triamcinolone with 5 fluorouracil (5FU) has gained popularity in recent years. Three milliliters of 5FU 50 mg/mL and 1 mL of the steroid containing 40 mg/mL triamcinolone make up the cocktail. For postoperative tissue infiltration at the level of the excision plane and below it, 1-2 mL of this mixture are employed. Once the wound has fully healed, the treatment can be resumed. After surgery, silicone gel should be used for two months, and postoperative pressure should be given for six months [16].

A systematic review and a meta-analysis explored various studies relating to the efficacy of surgical and silicone gel therapy for scar reduction, focusing on aspects such as hospital stay, postoperative pain, and scar reduction efficacy. Surgical excision of keloid scars, often followed by silicone gel sheeting, has shown a recurrence rate of 12.86%, with studies indicating the need for long-pressure applications across different body surfaces to achieve success. When combined with compression earring devices, the recurrence rate drops to 9.09%, with some patients experiencing temporary pruritus and pain resolving by the third postoperative week. The integration of silicone gel sheeting and compression earrings not only reduces scar recurrence but also minimizes postoperative discomfort. The therapeutic approach appears effective in preventing keloid formation, thus enhancing clinical outcomes. However, the duration of hospital stay and the detailed measurement of postoperative pain were not explicitly compared across the reviewed studies. Further research is required to standardize these parameters and verify the long-term efficacy and safety of combined therapies [17].

A systematic review by Bueno et al. [18] provides significant insights into the efficacy of various interventions in terms of postoperative pain and scar reduction efficacy. In terms of hospital stay, the study by Kong et al. [19] focused on patients undergoing bilateral total hip arthroplasty and compared standard wound closure with the use of tissue adhesive. The study found that, from the patient's perspective, the hips treated with tissue adhesive were significantly better than sutured hips, although no significant differences were noted in hospital stay durations. With respect to postoperative pain, Musham et al. [20] compared tissue adhesive and sub-cuticular sutures in thyroid surgery and found that patients in the tissue adhesive group experienced significantly less postoperative pain (p < 0.01). Additionally, the study by Karmisholt et al. [21] assessed the use of non-ablative fractional laser (NAFL) exposures and found that patients treated with NAFLs had better scores for relief and overall opinion (p = 0.037 and p = 0.003, respectively), suggesting reduced postoperative discomfort. On viewing the aspect of scar reduction efficacy, several studies highlighted significant improvements in scar quality with different interventions. Ilori et al. [22] demonstrated that microporous tape significantly improved scar height and width compared to the control group (p < 0.0001 each). Jensen et al. [23] found that the anti-CTFG (EXC001) treatment reduced scar severity significantly compared to placebo, with notable improvements in vascularity, pigmentation, thickness, and overall opinion by both physicians and patients (p < 0.001). Lin et al. [24] compared fractional CO2 laser treatments and found no significant differences in patient assessments, although physician assessments showed some improvement (p = 0.028). Overall, these studies suggest that interventions such as tissue adhesives, microporous tape, NAFLs, and anti-CTFG can significantly enhance scar quality, reduce postoperative pain, and potentially impact hospital stay, highlighting the importance of choosing the appropriate wound care strategy for optimal patient outcomes.

A study by Ueberschaer et al. [25] aimed to evaluate the functional and cosmetic outcomes of zigzag versus straight coronal incisions in neurosurgical patients. The postoperative pain was assessed indirectly through functional outcomes such as two-point discrimination, which was significantly better in the zigzag group (p = 0.005). This suggests that patients in the zigzag group experienced less nerve-related discomfort post-surgery. The study did not report direct measures of pain intensity or frequency. In terms of scar reduction efficacy, the zigzag incision demonstrated superior scar reduction efficacy compared to the straight incision. The width of the scar was significantly smaller in the zigzag group (p = 0.001). Additionally, scores on the Vancouver scar scale (VSS) and patient and observer scar assessment scale (POSAS) were significantly better in the zigzag group (p = 0.003 and p = 0.005, respectively), indicating better cosmetic outcomes and patient satisfaction. Overall, the zigzag incision led to better cosmetic and functional outcomes without additional details on hospital stay or direct postoperative pain levels being specified. These findings suggest a practice-changing potential for improving patient satisfaction and clinical results in neurosurgical procedures [25].

A comprehensive review evaluated the effectiveness of treatment methods for cesarean scar pregnancy (CSP), focusing on hospital stay, postoperative pain, and scar reduction efficacy. Hospital stay showed that patients treated with uterine artery chemoembolization (UAC) had shorter hospital stays compared to those treated with systemic methotrexate (MTX). On average, hospitalization lasted about nine days for UAC patients. This reduced hospital stay contributes to lower postoperative costs and faster patient recovery. Postoperative pain assessment showed that chemoembolization, performed under local anesthesia, combines chemotherapy with tissue ischemia, allowing a higher concentration of MTX to target the gestational foci for a longer period, resulting in fewer systemic toxic effects and less postoperative pain. The most commonly reported postoperative issue was lower abdominal pain, but it was significantly less severe compared to systemic treatments. Scar reduction efficacy measured indicates that the study emphasizes that UAC is highly effective for scar reduction. The efficacy rate for UAC ranges between 83% and 99%, with significant factors including gestational sac size and the presence of fetal heartbeat influencing the outcome. UAC treatment also results in quicker normalization of beta-human chorionic gonadotropin (b-hCG) levels, indicating successful treatment and reduced scarring. Compared to systemic MTX, UAC shows superior outcomes in reducing intraoperative blood loss and faster recovery, which directly contributes to better scar reduction. Overall, the study concludes that UAC is a superior treatment method for CSP in terms of reducing hospital stay, minimizing postoperative pain, and enhancing scar reduction efficacy compared to systemic MTX therapy [26].

Another study by Poelchow et al. [27] evaluated the efficacy of silicone gel compared to standard care for managing superficial partial thickness burns on the face and neck, focusing on scar reduction. In a single-blind, randomized controlled trial (RCT) involving 55 participants, the median time to healing was nine days for the silicone group and seven days for the control group, with no significant difference in healing time (p = 0.056). However, significant improvements were noted in scar pigmentation for the silicone group. At six weeks, the modified VSS (mVSS) scores showed significantly reduced pigmentation in the silicone group (median = 0, interquartile range (IQR) = 0) compared to the control group (median = 0, IQR = 0-3), with p = 0.043. Pain intensity scores did not differ significantly between the groups. Importantly, no adverse events were associated with silicone gel use, while the control group experienced an infection and a reaction. These results suggest that film-forming silicone gel effectively improves scar pigmentation in face and neck burns, highlighting its potential as a valuable treatment option for reducing scar formation in such injuries [27].

An RCT by Shen et al. [28] investigated the effectiveness of early eschar dermabrasion combined with antimicrobial soft silicone foam dressing in treating deep partial-thickness burn wounds in children. Following wound healing, both treatment groups - those receiving combined treatment and those receiving foam dressing alone - were administered a post-treatment protocol involving silicone gel application and elastic sleeve use to manage scars. After the wounds healed, children from both groups applied silicone gel twice daily for three weeks before transitioning to wearing elastic sleeves for more than 18 hours daily, continuing this regimen for over six months. The use of silicone gel, a well-known treatment for reducing scar formation, played a significant role in improving scar outcomes. Six months post-healing, the VSS was used to assess scar condition. The combined treatment group, which included the use of silicone gel, showed significantly better scar outcomes, with a median VSS score of 5 (range: 2-8), compared to the foam dressing group, which had a median VSS score of 7 (range: 5-10) (Z = -3.05, p < 0.05). This indicates that incorporating silicone gel into the treatment regimen contributed to more effective scar reduction, helping minimize scar hyperplasia and improve overall scar appearance in pediatric patients. Thus, silicone gel, combined with other treatments, proved to be an effective strategy for reducing scars in pediatric patients with deep partial-thickness burns, showcasing its potential to enhance healing outcomes when used consistently postinjury [28].

A systematic review by Sinha et al. [29] assessed various interventions for postburn pruritus, including surgical and silicone gel therapies. The review included 25 RCTs with 1,166 participants, evaluating 21 different interventions. The findings highlighted the use of physical modalities such as silicone gel sheeting as part of the therapeutic approaches for postburn pruritus. Silicone gel therapy, often used for scar management, was included in the interventions assessed. The review mentions enhanced education about silicone gel sheeting, a non-invasive approach known for its ability to hydrate the skin, reduce itchiness, and improve scar appearance. However, specific results from silicone gel use in this context were not detailed separately in the main results. It implies that silicone gel, as part of a broader category of topical therapies, may contribute to reducing pruritus when combined with other interventions. While the review covers surgical scar revision methods such as laser scar revision, specific surgical techniques targeting pruritus itself were not emphasized. Laser scar revision, which might be considered a surgical modality, showed moderate certainty evidence of reducing pruritus and pain. The pulsed high-intensity laser reduced pruritus intensity compared to placebo (MD: -0.51 on a 0-1 itch severity scale) and pain (MD: -3.23 on a VAS scale). Overall, while silicone gel therapy is recognized for its benefits in scar management, detailed evidence specific to its effectiveness in reducing postburn pruritus within this review is limited. Similarly, surgical methods such as laser revision showed potential but were discussed more broadly under laser therapies rather than traditional surgical procedures. The evidence across these interventions was considered moderate to low certainty due to the small size and high risk of bias in the included studies. Practitioners should carefully consider the applicability of these findings in clinical practice, given the varying certainty levels of the evidence [29]. This is shown in Table 1.

**Table 1:** Comparative studies on conventional scar management therapy Sources: [19-29]

Study	Intervention	Hospital Stay	Postoperative Pain	Scar Reduction Efficacy
Kong et al. [19]	Tissue adhesive vs. standard wound closure	No significant difference	Not specified	Tissue adhesive favored by patients
Musham et al. [20]	Tissue adhesive vs. sub-cuticular sutures in thyroid surgery	Not specified	Significantly less pain with tissue adhesive (p < 0.01)	Not specified
Karmisholt et al. [21]	Non-ablative fractional laser (NAFL)	Not specified	Better scores for relief and overall opinion (p = 0.037, p = 0.003)	Better scar outcomes with NAFL
Ilori et al. [22]	Microporous tape	Not specified	Not specified	Significant improvements in scar height and width (p < 0.0001)
Jensen et al. [23]	Anti-CTFG (EXC001) treatment	Not specified	Not specified	Significant improvements in vascularity, pigmentation, thickness, overall opinion (p < 0.001)
Lin et al. [24]	Fractional CO2 laser treatments	Not specified	Not specified	Some improvement in physician assessments (p = 0.028)
Ueberschaer et al. [25]	Zigzag vs. straight coronal incisions in neurosurgery	Not specified	Better 2-point discrimination in zigzag group (p = 0.005)	Superior scar reduction efficacy in zigzag group (VSS and POSAS scores)
Kłobuszewski et al. [26]	UAC vs. systemic MTX for cesarean scar pregnancy	Shorter for UAC (about 9 days)	Less severe pain with UAC	UAC more effective (efficacy rate 83-99%), faster beta-hCG normalization, reduced intraoperative blood loss
Poelchow et al. [27]	Silicone gel vs. standard care for superficial partial-thickness burns	No significant difference	No significant difference in pain intensity	Significant improvements in scar pigmentation with silicone gel (mVSS scores, p = 0.043)
Shen et al. [28]	Eschar dermabrasion + silicone gel in pediatric burns	Not specified	Not specified	Significantly better scar outcomes with silicone gel (VSS scores, p < 0.05)
Sinha et al. [29]	Various interventions for postburn pruritus	Not specified	Laser scar revision reduced pruritus and pain	Evidence varied, moderate to low certainty for silicone gel; laser scar revision showed potential

Laser Therapy

Laser therapy offers a minimally invasive, low-risk approach to treating pathological burn scars. It includes ablative carbon dioxide (CO_2_) lasers, NAFLs, and pulse dye lasers (PDLs). Ablative CO_2_ lasers target both the dermal and epidermal layers to reduce scar erythema, enhancing visibility. Non-ablative and fractional lasers focus on reducing scar thickness and volume by selectively damaging the dermis. PDLs use a lower wavelength of light absorbed by oxyhemoglobin to improve scar vascularity and appearance. While lasers are increasingly vital in burn scar management, treatment efficacy varies based on the laser type, wavelength, and optimal timing of therapy initiation [30-32].

A study by Haedersdal et al. [33] found that nonablative 1,540 nm fractional laser treatment significantly improved burn scar texture. Hospital stays were not directly affected by the laser treatments, as this therapy is an outpatient procedure. Postoperative pain was moderate and consistent across treatment sessions, with a median pain score of 5 on a scale of 0-10. Pain levels did not increase significantly despite higher fluences in subsequent sessions, possibly due to the laser's integrated cooling system and patient acclimatization to the procedure. Common postoperative side effects included erythema (redness), edema (swelling), bullae (blisters), and crusting, with erythema being universally observed immediately post-treatment. One patient experienced minor atrophic scarring as a side effect. In terms of scar reduction efficacy, the laser-treated areas showed marked improvements in texture, with significant reductions in unevenness compared to untreated control areas at both four and 12 weeks posttreatment. Skin texture scores improved from a baseline of 6 to 4 posttreatment, indicating smoother skin. Patients were generally satisfied with the results, with satisfaction scores remaining high throughout the follow-up period. Overall, the laser treatment demonstrated a promising potential for burn scar management with manageable side effects [33].

Similarly, a study by Lin et al. [34] on fractional photothermolysis for scar remodeling showed significant findings regarding hospital stay, postoperative pain, and scar reduction efficacy. Hospital stays were typically short, as the procedure is minimally invasive and performed on an outpatient basis, reducing the need for extended hospitalization. Regarding postopeative pain, most patients reported mild discomfort, which was manageable with over-the-counter pain medication. The study indicated that postoperative pain was generally lower with the non-ablative fractional photothermolysis (NAFR) compared to more invasive methods. In terms of scar reduction efficacy, the study demonstrated that NAFR significantly improved the appearance of hypertrophic scars. Patients in the low-density treatment arm (LDTA) showed more substantial improvement and fewer side effects than those in the high-density treatment arm (HDTA). At the three-month follow-up, patients in the LDTA group reported higher satisfaction and better overall scar appearance, with improvements in pigmentation, erythema, and texture. The study also suggested that younger scars (< 2 years) responded better to treatment than older scars (> 6 years). Overall, NAFR proved to be an effective and well-tolerated option for scar reduction, with the potential for early intervention yielding the best results [34].

Another study by Taudorf et al. [35] examined the efficacy of NAFL treatments on burn scars. Hospital stay specifics were not directly discussed, indicating that the study might have been outpatient-based. Postoperative pain was mild to moderate, with a median VAS score of 4 out of 10 across three treatment sessions, suggesting manageable pain levels during the treatment period. Scar reduction efficacy was significant. The treated scars showed substantial improvement in overall appearance, skin thickness, relief, and pliability compared to untreated areas. At six months, 88% of patients observed smoother scar texture in treated areas. Improvement was noted in both normotrophic and hypertrophic scars, though hypertrophic scars showed only mild to moderate improvement. Patient satisfaction remained stable throughout, with a median satisfaction score of 6 out of 10 at the final follow-up. Histological analysis revealed better skin architecture posttreatment, with more uniform collagen fibers and increased vascularization, aligning treated scars closer to normal skin structure. Adverse effects were minimal, including mild erythema and hyperpigmentation, with no significant adverse events impacting daily activities. Overall, NAFL treatments were effective in improving scar appearance and texture, with manageable pain and minimal side effects [35].

Supporting this, the findingsof Weshahy et al. study’s [36] on the efficacy of NAFL treatments for burn scars show significant improvements in various aspects. The laser-treated areas exhibited smoother and more pliable skin with better texture and thickness compared to untreated areas. Patients reported visual improvements in scar texture and were generally satisfied with the treatment outcomes. Hospital stay duration was not explicitly mentioned in the provided excerpts, suggesting that the treatments did not require extended hospitalization. Postoperative pain associated with the treatments was described as mild to moderate, with patients rating it between 2 and 7 on a 10-point scale. The pain was managed with cooling measures during the procedures. In terms of scar reduction efficacy, the study noted significant improvements in overall scar appearance, with a reduction in the modified POSAS (mPOSAS) scores from baseline to six months. Histological evaluations supported these findings, indicating collagen remodeling in treated scars. At six months, 88% of patients observed smoother skin in the treated areas, with varying degrees of improvement ranging from mild to considerable. Notably, scars with meshed skin grafts showed better responses to treatment compared to non-meshed skin. The study concluded that NAFL treatments are effective in inducing long-term clinical and histological improvements in mature burn scars, with manageable postoperative pain and no adverse effects worsening scar appearance [36].

Another study by Xi et al. [37] indicates that fractional laser treatments significantly improve scar texture and appearance, particularly for burn scars. Hospital stay duration was not directly addressed in the studies, but the focus on NAFLs suggests a preference for outpatient treatments with minimal downtime. Postoperative pain was reported to be minimal due to the intact epidermal barrier provided by NAFL, requiring little or no topical anesthetics and causing minor post-treatment discomfort. Scar reduction efficacy was evaluated through clinical and histological assessments. At six months posttreatment, 88% of laser-treated scars appeared smoother than untreated controls, with significant improvements in skin thickness, relief, and pliability. Patient satisfaction remained high throughout the postoperative period, with a median score of 6 out of 10 at the final follow-up. Histological analyses revealed collagen remodeling towards normal skin structure, supporting the clinical improvements observed. The use of fractional CO_2_ laser combined with growth factors was also found to be effective. Two months post-treatment, patients reported high satisfaction levels, and significant improvements were observed in scar pigmentation, pliability, vascularity, and height. These findings suggest that fractional laser treatments, particularly when combined with growth factors, provide substantial benefits in scar texture and appearance while maintaining a low pain profile and minimal recovery time [37].

Similarly, a study by Yang et al. [38] on the efficacy of pulsed dye laser (PDL) for treating hypertrophic scars postburn injury indicated significant scar reduction and manageable postoperative pain. The VAS was used to monitor pain, with average pain scores ranging from minimal to moderate across treatment groups. The VSS measured scar improvement, showing substantial enhancements in vascularity, pigmentation, pliability, and height of scars. Patients treated at different intervals (one, two, three, and four weeks) demonstrated notable VSS score improvements, with average reductions of 15%-30% in scar severity. Specifically, patients in the three-week interval group showed the most significant improvement, with a mean VSS score reduction of 25% (p < 0.01). Pain levels were generally well-tolerated, with no significant adverse events reported, making PDL a viable and effective option for managing hypertrophic scars [38].

In addition, a study on UltraPulse fractional CO2 laser treatment for extensive scarring after burns and trauma by Ge et al. [39] demonstrates significant efficacy in scar reduction and manageable postoperative pain, without extending hospital stays. Conducted on 21 patients with scars covering 20-65% of their total body surface area (TBSA), the treatment significantly improved scar texture, pliability, and pigmentation. The POSAS scores showed a notable decrease from an average of 70.03 to 55.03 (p = 0.002). Pain and pruritus scores also improved significantly, with pruritus reducing from 7.32 to 5.80 (p = 0.001). The treatment sessions, averaging 4.86 sessions per patient, did not result in any serious adverse events or functional impairments, maintaining a normal range of motion in all affected joints. Patient satisfaction was high, with an overall effectiveness rate of 100%, where 71.4% of patients reported very satisfactory results. The study supports the safety and effectiveness of UltraPulse fractional CO_2_ laser therapy for extensive scars, providing a viable option for significant scar reduction and pain management [39]. This is shown in Table 2.

**Table 2:** Comparative studies on laser therapy for scar management Sources: [33-39]

Study	Hospital Stay	Postoperative Pain	Scar Reduction Efficacy
Haedersdal et al. [33]	Outpatient, no extended stay	Moderate, median score 5/10	Significant texture improvement, reduced unevenness
Lin et al. [34]	Outpatient, minimal stay	Mild, manageable with OTC medication	Significant improvement in appearance, high satisfaction
Taudorf et al. [35]	Outpatient-based, no significant stay	Mild to moderate, median score 4/10	Significant improvement in texture, high satisfaction
Weshahy et al. [36]	Not explicitly mentioned	Mild to moderate, 2-7/10	Significant improvement in mPOSAS scores, collagen remodeling
Xi et al. [37]	Outpatient focus	Minimal, due to intact epidermal barrier	Significant texture and appearance improvement, high satisfaction
Yang et al. [38]	Not directly addressed	Minimal to moderate	Significant VSS score improvement, 15-30% reduction
Ge et al. [39]	No extended stay	Significant improvement, manageable	Significant improvement in texture, pliability, pigmentation, high satisfaction

Microneedling

A single-center, rater-blinded, split-face, placebo-controlled randomized clinical trial assessed the efficacy of a needling device for treating acne scars. Twenty healthy adults aged 20-65 were enrolled, and 15 completed the study. Participants underwent three needling treatments at two-week intervals. The treatment group showed significant scar reduction at six months (mean difference: 3.4; 95% CI: 0.2-6.5; p = 0.03) and nominal improvement at three months (mean difference: 2.4; 95% CI: -0.01 to 4.8; p = 0.052), whereas the control group showed no significant changes. The procedure was minimally painful (mean pain rating of 1.08 out of 10), and participants perceived a 41% improvement in scar appearance on the treated side. No adverse events were reported, indicating a safe and effective treatment with minimal discomfort and no hospital stay required [40].

Another study by Rana et al. [41] compared the efficacy of microneedling alone versus microneedling combined with 70% glycolic acid peels in treating atrophic acne scars. Sixty patients were randomized into two groups: Group 1 received microneedling at 0, 6, and 12 weeks, while Group 2 received the same microneedling schedule plus glycolic acid peels at 3, 9, and 15 weeks. A hospital stay was not required for either treatment, indicating both procedures are outpatient-based. Postoperative pain management details were not provided, but typically, such treatments involve minimal discomfort manageable with topical anesthetics. The study found that Group 2, receiving the combination treatment, showed a significantly greater reduction in mean ECCA scores compared to Group 1 (39.65±2.50 vs. 29.58±0.18; p < 0.001), indicating better scar improvement. Additionally, Group 2 reported more significant improvements in skin texture on the VAS. Thus, adding 70% glycolic acid peels to microneedling enhances scar reduction and improves skin texture more effectively than microneedling alone [41].

Another study by Ali et al. [42] investigated the efficacy of microneedling combined with Jessner’s solution peeling for treating atrophic acne scars. In view of hospital stay, the treatment involved microneedling and chemical peeling procedures, which are typically performed on an outpatient basis. Postoperative pain was managed effectively in the study. The application of local anesthetic cream before the procedure minimized discomfort. After the treatment, cold packs were applied to reduce immediate pain and swelling. There were no significant reports of postoperative pain that required extended medical intervention. The combined treatment of microneedling and Jessner’s solution peeling demonstrated significant improvement in the appearance of atrophic acne scars. The study categorized clinical efficacy into four levels: very significant (> 75-100% improvement), marked (> 50-75% improvement), moderate (> 25-50% improvement), and mild (< 25% improvement). The results showed that patients who received the combined treatment experienced better scar reduction compared to those who received either treatment alone. Most patients observed a notable decrease in scar severity, with the greatest improvements seen in patients with Grade 2 and 3 scars. The combined approach was particularly effective in reducing post-inflammatory hyperpigmentation and enhancing overall skin texture and appearance. In summary, the study found that the combination of microneedling and Jessner’s solution peeling is an effective outpatient treatment for atrophic acne scars, with minimal postoperative pain and significant improvements in scar reduction [42].

 A study by An et al. [43] focused on combination therapy with topical poly-lactic acid and microneedle fractional radiofrequency and did not report any hospital stay requirements for the treatments. Both combination therapy and monotherapy were outpatient procedures, indicating minimal to no hospital stay. Postoperative pain was not specifically quantified in this study. However, both treatments being outpatient and non-invasive suggest minimal postoperative pain. The study focused more on patient satisfaction, which indirectly reflects tolerability and comfort post-treatment. Combination therapy with topical poly-lactic acid and MFRF showed significantly better outcomes compared to MFRF with normal saline. Visual assessment and patient satisfaction scores were significantly higher with combination therapy (p = 0.036 and p = 0.009, respectively). Scar smoothness (p < 0.001), scar size (p = 0.003), and overall improvement (p < 0.001) were significantly better in the combination therapy group, although there was no significant difference in scar brightness (p = 0.151). While monotherapy with MFRF showed improvements, it was less effective compared to the combination therapy, indicating the added benefit of using poly-lactic acid in enhancing scar treatment efficacy [43].

A clinical trial compared topical tazarotene gel and microneedling therapy. Similar to prior studies, both treatments were conducted on an outpatient basis, indicating no hospital stay required. Patients could perform the topical treatment at home, and microneedling sessions were done periodically without necessitating hospitalization. The study did not explicitly measure postoperative pain but monitored patient satisfaction and comfort. The treatments were well-tolerated by participants, with no major adverse effects reported, suggesting minimal postoperative discomfort. The application of 0.1% tazarotene gel once nightly showed significant improvement in acne scar severity. The median quantitative score for acne scar severity improved from 8.0 (6.0-9.8) to 5.0 (3.0-6.0) at the six-month follow-up (p < 0.001), demonstrating the efficacy of the topical treatment. Microneedling with a 1.5 mm derma roller also resulted in significant improvement. The median quantitative score improved from 7.0 (6.0-10.8) to 4.5 (3.0-6.0) (p < 0.001). Both treatments yielded comparable results for scar severity reduction (p = 0.42). The qualitative acne scar scores did not show a significant difference between the two treatments, indicating that both treatments were equally effective in reducing acne scar severity [44]. In conclusion, both studies highlighted the efficacy of combination therapies and standalone treatments in reducing acne scars with minimal hospital stay and postoperative pain, emphasizing the importance of personalized treatment plans for optimal results. This is shown in Table 3.

**Table 3:** Comparative studies for microneedling in scar reduction Sources: [40-44]

Study	Design	Postoperative Pain	Scar Reduction Efficacy	Additional Notes
Alam et al. [40]	Rater-blinded, split-face, placebo-controlled	Mean pain rating: 1.08/10	Significant scar reduction at 6 months (mean difference: 3.4; 95% CI, 0.2-6.5; p = 0.03)	41% perceived improvement, no adverse events
Rana et al. [41]	Randomized clinical trial	Not specifically reported	Combination group (microneedling + 70% glycolic acid) showed greater reduction in ECCA scores (39.65±2.50 vs. 29.58±0.18; p < 0.001)	Combination therapy improved skin texture more significantly
Ali et al. [42]	Comparative study	Managed with local anesthetics and cold packs	Significant improvement, particularly in grade II and III scars	Combined treatment better than individual treatments, effective in reducing post-inflammatory hyperpigmentation
An et al. [43]	Randomized clinical trial	Not specifically quantified, minimal expected	Combination therapy showed better outcomes in scar smoothness (p < 0.001), scar size (p = 0.003), and overall improvement (p < 0.001)	No significant difference in scar brightness (p = .151)
Afra et al. [44]	Prospective, observer-blinded, randomized clinical trial	Not explicitly measured, minimal reported	Tazarotene: median quantitative score improved from 8.0 to 5.0; Microneedling: from 7.0 to 4.5 (p < 0.001)	Both treatments equally effective (P = .42)

A systematic review by Mujahid et al. [45] provides detailed findings on microneedling in the treatment of acne scars. In terms of hospital stay, microneedling has a notably shorter recovery period compared to other treatments. One study reported an average downtime of 1.47 ± 0.57 days for microneedling, while fractional erbium-doped yttrium aluminum garnet laser treatment required about 5.07 ± 0.69 days of downtime. On viewing the postoperative pain, patients undergoing microneedling experience significantly less pain compared to those receiving laser treatments. Microneedling scored lower on pain scales compared to lasers in multiple studies. However, when compared to fractional microneedling combined with bipolar radiofrequency, microneedling resulted in more erythema, pain, and edema. In view of scar reduction efficacy, microneedling showed high efficacy in scar reduction, often enhanced when combined with other treatments. Studies indicate that microneedling combined with glycolic acid or trichloroacetic acid yielded more significant scar improvement than microneedling alone. In a clinical trial, the combination therapy group exhibited better outcomes than the monotherapy group. The Goodman and Baron Qualitative Scale and other scoring systems consistently show improvement in scar severity with microneedling treatments. Overall, microneedling offers a shorter hospital stay and reduced postoperative pain while effectively improving scar appearance, especially when used with complementary treatments. The document underscores the promising nature of microneedling as a well-tolerated and effective method for acne scar treatment [45].

Platelet-Rich Plasma (PRP)

PRP has shown promise in scar reduction and post-operative management due to its growth factors and bioactive components. PRP treatments for traumatic scars, acne scars, and post-procedure recovery have demonstrated significant improvements.

In scar revision, PRP combined with fat grafting has prolonged fat survival by up to a year and improved scar aesthetics according to the Manchester Scar Scale. Combining PRP with nonablative laser treatments has shown notable enhancements in scar texture, color, and overall appearance. However, some studies reported qualitative results without clear quantitative data, making conclusive analysis difficult. For acne scars, PRP has been evaluated in combination with fractional laser therapies. Topical PRP gel application post-laser showed over 50% improvement in most patients. Split-face studies using L-PRP injections post-CO2 laser therapy revealed reduced erythema duration and improved clinical appearance. Both topical and intradermal L-PRP applications resulted in significant acne scar improvements and shorter recovery times compared to laser therapy alone. Optical coherence tomography confirmed deeper scar reduction with PRP treatments. Post-procedure recovery with PRP application, particularly after rhytidectomy and fractional laser treatments, resulted in reduced erythema, melanin indices, and transepidermal water loss. PRP-treated areas also exhibited thicker collagen bundles, indicating enhanced healing and skin quality. Overall, PRP enhances scar reduction and post-operative recovery by promoting tissue regeneration, reducing inflammation, and improving aesthetic outcomes, making it a valuable adjunct in dermatological and cosmetic procedures [10].

A study by Heitmiller et al. [46] aimed to compare the efficacy and safety of carboxytherapy and PRP injections in treating striae alba. Twenty patients received PRP injections on the right side (group A) and carboxytherapy on the left side (group B) every three to four weeks for a total of four sessions. Skin biopsies taken before and after treatment were analyzed using fibronectin immunohistochemical staining. Results showed significant improvement in striae alba in both groups posttreatment compared to pretreatment levels. The study found no significant difference between the two treatment modalities regarding percentage improvement, response on a grading scale, or patient satisfaction. However, the area stained with fibronectin was significantly higher posttreatment in both groups, indicating increased fibronectin expression, a marker for tissue repair and regeneration. Interestingly, group B (carboxytherapy) showed a significantly higher fibronectin expression than group A (PRP). Both treatments were deemed safe with minimal side effects, and no notable differences were observed in terms of hospital stay or postoperative pain between the two groups. The efficacy of scar reduction was comparable between the PRP and carboxytherapy treatments, with both methods effectively enhancing fibronectin expression and improving the appearance of striae alba. This histopathological improvement suggests that both modalities can be valuable options for treating striae distensae (SD), although carboxytherapy may offer a slight edge in enhancing fibronectin expression [46].

Interestingly, a study by Suwanchinda et al. [47] evaluated the efficacy and safety of innovative cold atmospheric plasma (CAP) technology for treating SD in 23 patients. The body was divided into two halves, with one side receiving biweekly CAP treatments for five sessions, while the other side remained untreated. A hospital stay was not required, indicating outpatient treatment. Postoperative pain was minimal, with adverse effects such as small scabs, shallow wounds, and rash being the only noted issues. These side effects were minor and managed easily, with pain assessment using the VAS indicating low discomfort. Scar reduction efficacy was significant. The treated areas showed a statistically significant reduction in all parameters on the POSAS compared to untreated areas (p-value < 0.05). Patient satisfaction was high, with 52.3% reporting great improvement, 39.1% moderate improvement, 4.3% extreme improvement, and 4.3% slight improvement. Antera 3D® skin imaging confirmed these findings, demonstrating substantial improvement in the treated areas. This study thus showed that CAP is an effective and safe treatment for SD, providing notable scar reduction and high patient satisfaction with minimal side effects [47].

Similarly, a study by Ibrahim et al. [48] included 68 patients who were divided into three groups: Group I received an intradermal injection of PRP, Group II underwent microdermabrasion, and Group III received a combination of both treatments. The hospital stay was not explicitly mentioned, implying outpatient procedures. Postoperative pain was a common side effect, particularly in PRP-treated groups. In Group I, 46.7% of patients reported pain during injection, and in Group III, 80% experienced pain. In contrast, Group II had no post-therapy complaints, indicating that microdermabrasion was better tolerated. Scar reduction efficacy was evaluated through patient satisfaction and clinical improvement. Group I showed a significant increase in skin texture improvement, with 30.4% of patients very satisfied. Group III, which combined PRP and microdermabrasion, showed the highest satisfaction, with 36.4% of patients very satisfied and the least number of sessions required for clinical improvement. Histopathological examination revealed enhanced collagen deposition and elastic fiber arrangement in PRP-treated groups, suggesting better scar reduction efficacy compared to microdermabrasion alone. In summary, PRP, both alone and combined with microdermabrasion, was effective in reducing scars, though it came with manageable postoperative pain. Microdermabrasion alone was less effective in scar reduction but had fewer side effects, making it a more comfortable option for patients. Further research is needed to confirm these findings and optimize treatment protocols [48].

Despite the increasing application of PRP in scar management, evidence supporting its efficacy is limited. A systematic review and meta-analysis of randomized clinical trials up to September 1, 2020, was conducted to evaluate the effectiveness and safety of PRP for atrophic and hypertrophic/keloidal scars. Thirteen clinical trials were included in the meta-analysis, with 10 additional studies reviewed. The overall response rate for PRP-treated patients was 23%, similar to laser or micro-needling treatments (22% and 23%, respectively). When used alone, PRP resulted in moderate improvement in 36% of cases. However, when combined with laser or micro-needling, patients experienced marked improvement (33% and 43%, respectively) and excellent results (32% and 23%, respectively). For hypertrophic/keloid scars, the single qualifying study showed better improvement and fewer adverse effects when PRP was added to intralesional corticosteroids [49].

Overall, PRP is a safe and effective treatment for various atrophic scars and enhances the efficacy of ablative lasers or micro-needling, reducing side effects. The addition of PRP to other treatments provides significant benefits in scar reduction and patient outcomes.

Stem Cell Therapy

Mesenchymal stem cell (MSC) therapy has emerged as a promising treatment for burn injuries and scar reduction. The first successful transformation of bone marrow-derived MSCs (BM-MSCs) in 2005 demonstrated their safety and efficacy in a 45-year-old female with extensive burns. This treatment facilitated new vessel formation, rapid granulation, and reduced scar formation. Subsequent studies, including the use of cadaveric BM-MSCs, showed significant improvements in wound healing, reduced pain, and enhanced tissue regeneration. Cell therapy, particularly with fibroblast-like MSCs (FMSCs), has shown significant promise in treating severe burn injuries. Initially, treatments involved the use of allofibroblasts and keratinocytes, which, despite their effectiveness, proved too costly for widespread use. The application of fetal fibroblasts demonstrated improved wound regeneration and reduced mortality, but ethical and legal concerns limited their practicality. Consequently, BM-MSCs became a focus, given their ability to differentiate into various cell types, including fibroblast-like cells. A case study of a 45-year-old female with extensive burns (40% total body surface, 30% IIIB degree) illustrated the efficacy of allogenic FMSC therapy. Following conventional treatments and multiple necrectomies, FMSC transplantation was performed, leading to enhanced granulation, new vessel formation, and pain relief. Skin grafts were successfully adhered, and the patient's condition improved markedly, with significant epithelialization and reduced plasmarrhea. The therapy facilitated quicker and more effective skin grafting, improving overall healing outcomes [50].

A study by Roohaninasab et al. [51] evaluated the efficacy and safety of using subcision combined with stromal-vascular fraction (SVF) versus subcision alone in treating acne scars. In this double-blind clinical trial involving 10 patients, one side of the face received subcision plus SVF, while the other side underwent subcision only. Results showed that the combined therapy significantly reduced scar volume and area, with mean percent changes of 46.55% and 44.60%, respectively, compared to 13.31% and 11.28% for subcision alone (p < 0.001). The combined treatment also increased the density and thickness of the epidermis and dermis more effectively. Both doctor and patient satisfaction scores were higher for the combined therapy (7.10 vs. 5.50 and 5.30, respectively). No complications were reported. Thus, the combined approach proved to be more effective and safe for acne scar reduction, offering superior results in scar improvement and patient satisfaction without additional postoperative complications [51].

Another study by Xu et al. [52] investigated the use of autologous BM-MSCs to improve the quality of wound healing and reduce hypertrophic scar formation in a 19-year-old male burn patient. The patient had severe burns covering over 80% of his body, resulting in significant hypertrophic scarring. BM-MSCs were cultured and injected into split-skin graft sites on one arm, while a control site on the other arm received only a split-skin graft without BM-MSCs. Postoperative outcomes were positive, with complete wound healing and no pain or infection at both sites. However, at one-year and two-year follow-ups, the BM-MSC-treated site showed significantly less contraction and better overall appearance compared to the control site. The BM-MSC site had smoother, more extensive skin coverage and fewer complications related to hypertrophic scarring. Hospital stay duration, although not explicitly stated, was inferred to be beneficially impacted due to improved healing and reduced complications. The study highlighted BM-MSCs' potential to decrease skin graft contraction and reduce scar-related issues, suggesting the need for larger clinical trials to confirm these promising results and understand the underlying mechanisms [52].

Similarly, a study by Yoshikawa et al. [53] introduced a novel wound treatment using cultured marrow mesenchymal cells (MMCs) combined with an artificial dermis made of collagen sponge. This method was tested on 20 patients with intractable skin wounds, demonstrating its efficacy in tissue regeneration. Marrow mesenchymal cells from a 46-year-old donor were cultured and placed into a collagen sponge, which was then implanted in a nude mouse. Immuno-histological analysis confirmed tissue regeneration using human MMCs. Subsequently, 10-20 mL of bone marrow fluid was aspirated from the patients, cultured, and combined with the collagen sponge for wound treatment. Out of the 20 patients (average age 64.8 years), 18 experienced significant wound healing, while two patients died from unrelated causes. The study highlighted the therapeutic effectiveness of autologous MMC transplantation, which promoted skin regeneration with minimal invasiveness compared to traditional skin grafting techniques. The minimally invasive nature of bone marrow aspiration reduced postoperative pain and shortened hospital stays. The technique also effectively reduced scar formation, improving clinical outcomes for patients with chronic dermatopathies. This innovative approach offers a practical and less invasive alternative to existing skin grafting methods, showing promise for broader clinical application [53].

Interestingly, a study by Rasulov et al. [54] pioneered the use of cadaveric bone marrow MSCs (CMSCs) for treating deep skin burns. The subject, a young man who suffered severe burns over 60% of his body, including 30% full-thickness burns, received CMSCs derived from a cadaver donor. These stem cells were cultured, expanded, and applied to the burned areas using a fibrin spray following early escharotomy. Preliminary results have shown impressive safety and efficacy, with notable improvements in wound healing, reduced hospital stay, and decreased postoperative pain. The application of CMSCs led to significant scar reduction, contributing to better functional and cosmetic outcomes compared to traditional treatments. The technique demonstrated the potential to minimize pain and accelerate recovery, thereby reducing the overall duration of hospitalization. The study underscores the promise of CMSCs in regenerative medicine, particularly for burn patients, and suggests that this approach could revolutionize treatment protocols by saving time and lives. The early success of CMSCs in this case suggests a significant scientific opportunity for broader applications in regenerative medicine and transplantation, encouraging further research and development in this field [54].

Botulinum

A study by Rasaii et al. [55] compared intralesional triamcinolone alone and in combination with botulinum toxin in treating keloidal scars in 20 patients with 40 keloids. Results indicated no significant difference between the treatments regarding vascular score, flexibility, and pigmentation. However, the addition of botulinum toxin significantly improved symptomatic pain and pruritus compared to triamcinolone alone. This suggests that, while both treatments are effective in general scar characteristics, botulinum toxin may offer additional benefits in symptom management. In a randomized clinical trial by Shaarawy et al. [56], 24 keloid patients were divided into two groups: one receiving intralesional steroids every four weeks and the other receiving botulinum toxin (BTA) every eight weeks. Both treatments significantly reduced lesion volume and complaints such as itching, pain, and tenderness. However, BTA showed more pronounced improvement in patient complaints and fewer side effects. Skin atrophy and telangiectasia were noted in the steroid group, indicating that BTA is more effective and safer than corticosteroids for keloid treatment. A prospective clinical study by Elhefnawy et al. [57] involved 20 patients with hypertrophic ulcers treated with monthly intralesional BTA injections for three months and followed for six months. Significant improvements were observed in erythema, pruritus, and flexibility scores, all statistically significant. Patient satisfaction was high, with 14 reporting "good" and six reporting "excellent" outcomes. These findings underscore BTA's efficacy in improving scar appearance and reducing symptoms. Another study by Zhibo et al. [58] involving 12 patients with keloids received BTA injections every three months over a nine-month period. Follow-up for one year showed varied treatment responses: five patients had a good response, four had an average response, and three had an excellent response, with no recurrences reported. Significant reductions were seen in redness, consistency, and pruritus scores. The study concluded that BTA is effective for keloid treatment, although further research is needed to confirm these results.

A clinical trial investigated the effects of BTA injection on scar formation in 45 patients with forehead ulcers. Twenty-four patients received BTA injections, while 21 received normal saline. Assessments using POSAS and VAS scales at one, three, and six months showed significant improvement in the BTA group. Skin biopsies revealed less collagen storage in the BTA-treated group, indicating aesthetic, functional, and psychological benefits. Therefore, BTA injections can be effective in preventing hypertrophic scars following burns, trauma, or surgery [59]. In a study by Ziade et al. [60], the early injection of BTA was examined for its effect on facial ulcers in 30 patients. Patients were divided into control and intervention groups, with BTA injected 72 hours postsurgery. After one year, 24 patients were assessed using POSAS, VSS, and VAS scales. While POSAS and VSS scores showed no significant differences, the VAS score was significantly higher in the intervention group (8.25) compared to the control group (6.35). This suggests that BTA injections can enhance facial wound healing by reducing pressure lines on the skin. Another clinical trial assessed the efficacy of BTA injection for epicanthoplasty scars in 43 patients. BTA or saline was injected six to seven days postsurgery, and lesions were evaluated at one, three, and six months using VAS and VSS scales. The BTA-treated group showed significant improvement in VSS scores, particularly in height and flexibility, with the greatest improvement at three months. The VAS score also significantly decreased, with 86.7% of patients highly satisfied. Early BTA treatment following epicanthoplasty reduces hypertrophic scars and enhances surgical outcomes [61]. Another study by Lee et al. [62] combined 595 nm PDL therapy with intramuscular BTA injection to treat traumatic chin scars in two patients. Both patients achieved good aesthetic results, with only mild pain during and after treatment, resolving within days. This combination treatment proved to be safe and effective for trauma-induced chin scars. Similarly, in a double-blind, randomized clinical trial, 60 patients were divided into intervention and control groups. The intervention group received BTA injections at the orbicularis oris muscle site post-surgery, while the control group received saline. After six months, the intervention group showed a significant reduction in lesion width and improved VAS scores. However, VSS scores were similar between groups. Thus, BTA injections improve scar appearance and width but do not affect pigmentation, vascularity, flexibility, or height [63].

Discussion

The results of the conventional therapies underscore the effectiveness of various techniques and therapies for scar management, emphasizing their impact on scar appearance, recurrence, postoperative pain, and hospital stay. Techniques such as layered wound repair, appropriate suture sizes, and fusiform elliptical excision are noted for their ability to enhance the strength and aesthetic outcome of repairs, particularly in alignment with relaxed skin tension lines (RSTLs). Z-plasty and its variations (e.g., multiple Z-plasties, double opposing Z-plasties) are highlighted for addressing webbed or contracted scars, redistributing tension, and improving functional outcomes. Dermabrasion and microdermabrasion offer additional options to smooth and blend scar surfaces with the surrounding skin, while advanced interventions such as flaps, grafts, and surgical debulking are reserved for large, complex scars. The combined use of triamcinolone and 5FU is emerging as a popular postoperative treatment for keloid scars, significantly reducing recurrence when applied appropriately. Studies indicate that combining surgical excision with silicone gel sheeting and compression devices effectively minimizes keloid scar recurrence, reducing it to 9.09%. Silicone gel sheeting, in particular, improves scar pigmentation and reduces postoperative discomfort, making it a valuable adjunct therapy. The use of silicone gel post-treatment, as shown in studies involving pediatric burn patients, also contributes significantly to scar reduction, demonstrating better VSS scores. Additionally, tissue adhesives and microporous tape are found to enhance scar quality and reduce postoperative pain compared to traditional sutures. NAFLs and anti-CTFG treatments further improve scar outcomes by reducing thickness, pigmentation, and vascularity. These findings advocate for a multidisciplinary approach to scar management, combining surgical techniques with postoperative therapies such as silicone gel sheeting and compression devices to achieve optimal cosmetic and functional outcomes. The evidence also suggests that choosing the appropriate intervention tailored to the scar's characteristics and patient needs can significantly improve patient satisfaction and clinical results.

The studies in laser therapy demonstrate the efficacy and advantages of laser therapy in managing burn scars. Ablative CO_2_ lasers, NAFLs, and pulsed dye lasers (PDLs) are effective in improving scar texture, thickness, and appearance, with manageable postoperative pain and minimal recovery time. The non-ablative 1,540 nm fractional laser showed significant texture improvements and high patient satisfaction, with manageable pain scores of 5 out of 10 and common side effects such as erythema and edema. Fractional photothermolysis proved effective for hypertrophic scars, particularly in younger scars, offering minimal discomfort and short hospital stays due to its outpatient nature. NAFL treatments were highlighted for their effectiveness in improving scar pliability and texture, with high patient satisfaction and mild to moderate pain. Studies showed significant reductions in scar severity, improved skin architecture, and high overall effectiveness rates. PDL treatments were effective in reducing hypertrophic scar severity, with manageable pain and notable improvements in vascularity, pigmentation, and pliability. Combining laser treatments with growth factors or silicone gel further enhanced scar outcomes, supporting long-term clinical and histological improvements. Overall, these studies underscore the advantages of laser therapy in burn scar management, including minimal invasiveness, low postoperative pain, high patient satisfaction, and significant improvements in scar appearance and texture.

The reported studies on microneedling techniques highlight the effectiveness and advantages of microneedling and combination therapies for treating acne scars, particularly focusing on outpatient procedures with minimal hospital stay and postoperative pain. A single-center, rater-blinded, split-face, placebo-controlled randomized clinical trial demonstrated significant scar reduction with a needling device, with participants experiencing minimal pain and no adverse events, indicating a safe and effective treatment. Rana et al.'s [41] study showed that combining microneedling with 70% glycolic acid peels significantly improved scar reduction compared to microneedling alone, with greater patient satisfaction and skin texture improvements. Similarly, Ali et al. [42] found that microneedling combined with Jessner’s solution peeling effectively reduced atrophic acne scars, particularly for grade II and III scars, with manageable postoperative pain and notable enhancements in skin texture and appearance. An et al.'s [43] research on combination therapy with topical poly-lactic acid and microneedle fractional radiofrequency revealed better outcomes than monotherapy, emphasizing the added benefits of poly-lactic acid in enhancing scar treatment efficacy. A clinical trial comparing topical tazarotene gel and microneedling therapy showed significant improvements in acne scar severity, with both treatments being equally effective and well-tolerated by participants. Lastly, a systematic review by Mujahid et al. [45] highlighted microneedling's shorter recovery period and reduced postoperative pain compared to laser treatments, emphasizing its high efficacy in scar reduction, especially when combined with other treatments such as glycolic acid or trichloroacetic acid. Overall, these studies underscore microneedling's efficacy and tolerability, making it a promising option for acne scar management.

PRP and stem cell therapy have emerged as promising treatments for scar reduction and postoperative management due to their regenerative properties. PRP, rich in growth factors and bioactive components, has shown significant improvements in traumatic scars, acne scars, and post-procedure recovery. Studies combining PRP with fat grafting or nonablative laser treatments report enhanced scar aesthetics, texture, color, and overall appearance. For acne scars, PRP combined with fractional laser therapies has demonstrated over 50% improvement, reduced erythema duration, and quicker recovery times. Optical coherence tomography and histopathological examinations confirm deeper scar reduction and thicker collagen bundles in PRP-treated areas, underscoring its efficacy in promoting tissue regeneration and reducing inflammation.

Stem cell therapy, particularly using MSCs, has shown significant promise in treating severe burn injuries and reducing scar formation. MSCs facilitate new vessel formation, rapid granulation, and enhanced tissue regeneration, improving overall healing outcomes. Studies using BM-MSCs demonstrate significant reductions in scar contraction and hypertrophic scarring, with better skin appearance and fewer complications. Novel approaches, such as combining MSCs with collagen sponges or using cadaveric MSCs, have shown impressive results in wound healing, reduced hospital stays, and minimal postoperative pain. These therapies offer minimally invasive alternatives to traditional treatments, emphasizing their potential in regenerative medicine and scar management.

Botulinum toxin (Botox) has demonstrated potential in treating keloidal and hypertrophic scars by reducing symptomatic pain, pruritus, and improving overall scar appearance. Studies have shown that, while Botox combined with intralesional steroids does not significantly affect the vascular score, flexibility, or pigmentation compared to steroids alone, it notably enhances symptom management, particularly pain and itchiness. Botox is also effective as a standalone treatment, reducing erythema and pruritus and improving scar flexibility with high patient satisfaction. Clinical trials indicate that Botox injections can prevent hypertrophic scars following burns, trauma, or surgery by reducing collagen deposition and pressure lines on the skin, leading to improved aesthetic and functional outcomes. Additionally, combining Botox with laser therapies has shown promising results in treating traumatic scars, providing good aesthetic outcomes and minimal side effects. Overall, Botox offers a safe and effective adjunctive treatment for scar management, enhancing patient satisfaction and quality of life.

## Conclusions

In conclusion, the efficacy of various scar reduction techniques has been significantly demonstrated across multiple studies, emphasizing the critical aspects of hospital stay duration, postoperative pain management, and scar reduction quality. Techniques such as the use of tissue adhesives, microporous tape, NAFL, and anti-CTFG treatments have shown substantial improvements in reducing postoperative pain and enhancing scar quality. For instance, patients treated with tissue adhesive experienced significantly less postoperative pain compared to those treated with sub-cuticular sutures, with similar hospital stay durations. Moreover, interventions such as NAFL and anti-CTFG treatments have been shown to provide better scores for relief and overall patient satisfaction, indicating a significant reduction in postoperative discomfort. In terms of scar reduction efficacy, studies have highlighted the benefits of microporous tape, which significantly improved scar height and width, and anti-CTFG treatment, which showed notable improvements in vascularity, pigmentation, and thickness of scars. Additionally, the zigzag incision technique in neurosurgical procedures has demonstrated superior cosmetic and functional outcomes compared to straight incisions, further reducing scar severity and improving patient satisfaction. These findings underscore the importance of selecting appropriate wound care strategies to achieve optimal patient outcomes. By effectively reducing hospital stays, minimizing postoperative pain, and enhancing scar quality, these advanced interventions not only improve clinical results but also significantly enhance patient quality of life and satisfaction.

## References

[1] Al-Shaqsi S, Al-Bulushi T (2016). Cutaneous scar prevention and management: overview of current therapies. Sultan Qaboos Univ Med J.

[2] Arno AI, Gauglitz GG, Barret JP, Jeschke MG (2014). Up-to-date approach to manage keloids and hypertrophic scars: a useful guide. Burns.

[3] Baryza MJ, Baryza GA (1995). The Vancouver scar scale: an administration tool and its interrater reliability. J Burn Care Rehabil.

[4] Draaijers LJ, Tempelman FR, Botman YA, Tuinebreijer WE, Middelkoop E, Kreis RW, van Zuijlen PP (2004). The patient and observer scar assessment scale: a reliable and feasible tool for scar evaluation. Plast Reconstr Surg.

[5] Durani P, McGrouther DA, Ferguson MW (2009). Current scales for assessing human scarring: a review. J Plast Reconstr Aesthet Surg.

[6] Gold MH, Berman B, Clementoni MT, Gauglitz GG, Nahai F, Murcia C (2014). Updated international clinical recommendations on scar management: part 1--evaluating the evidence. Dermatol Surg.

[7] Watson D, Reuther MS (2012). Scar revision techniques-pearls and pitfalls. Facial Plast Surg.

[8] Camirand A, Doucet J (1997). Needle dermabrasion. Aesthetic Plast Surg.

[9] Orentreich DS, Orentreich N (1995). Subcutaneous incisionless (subcision) surgery for the correction of depressed scars and wrinkles. Dermatol Surg.

[10] Leo MS, Kumar AS, Kirit R, Konathan R, Sivamani RK (2015). Systematic review of the use of platelet-rich plasma in aesthetic dermatology. J Cosmet Dermatol.

[11] Wang M, Xu X, Lei X, Tan J, Xie H (2021). Mesenchymal stem cell-based therapy for burn wound healing. Burns Trauma.

[12] Dilmaghani S, Behrangi E, Mazandarani M, Pourali A, Sadeghi S, Khosravi M, Goodarzi A (2022). Needling, lasers, and meso-botox for hypertrophic and keloidal scars: a comprehensive review study on promising procedural treatments. J Family Med Prim Care.

[13] Goodman GJ (2010). The use of botulinum toxin as primary or adjunctive treatment for post acne and traumatic scarring. J Cutan Aesthet Surg.

[14] Kim J (2021). Topographic computer analysis for acne scar treatment on face accompanying biopsy study after dermal injection of hydrotoxin mixture. J Cosmet Dermatol.

[15] Garg S, Dahiya N, Gupta S (2014). Surgical scar revision: an overview. J Cutan Aesthet Surg.

[16] Chambers A (2020). Management of scarring following aesthetic surgery. Textbook on Scar Management: State of the Art Management and Emerging Technologies.

[17] Tahir SM, Ihebom D, Simman R (2024). Compression therapy for keloid scars: a systematic review and meta-analysis. Plast Reconstr Surg Glob Open.

[18] Bueno A, Nevado-Sanchez E, Pardo-Hernández R, de la Fuente-Anuncibay R, González-Bernal JJ (2023). Treatment and improvement of healing after surgical intervention. Healthcare (Basel).

[19] Kong X, Yang M, Cao Z, Chen J, Chai W, Wang Y (2020). Tissue adhesive for wound closure in enhanced-recovery total hip arthroplasty: a prospective, randomized and controlled study. BMC Musculoskelet Disord.

[20] Musham A, Samuel EM, Sahoo AK, Elamurugan TP, Manwar AS (2023). Comparison of tissue adhesive glue with subcuticular absorbable suture for skin closure following thyroid surgery: a single-blinded randomised controlled trial. Sultan Qaboos Univ Med J.

[21] Karmisholt KE, Banzhaf CA, Glud M, Yeung K, Paasch U, Nast A, Haedersdal M (2018). Laser treatments in early wound healing improve scar appearance: a randomized split-wound trial with nonablative fractional laser exposures vs. untreated controls. Br J Dermatol.

[22] Ilori OS, Oladele AO, Ilori OR, Onilede DA (2022). Efficacy of microporous tape in the prevention of abnormal post-surgical scars among a black population. J Ayub Med Coll Abbottabad.

[23] Jensen J, Gentzkow G, Berman G (2018). Anti-CTGF oligonucleotide reduces severity of postsurgical hypertrophic scars in a randomized, double-blind, within-subject, placebo-controlled study. Plast Reconstr Surg.

[24] Lin MJ, Dubin DP, Torbeck RL 3rd (2023). Early fractional ablative laser for skin cancer excision scars: a randomized split-scar study. Dermatol Surg.

[25] Ueberschaer M, Endres M, Wachtel N (2023). A prospective randomized comparison of functional and cosmetic outcomes of a coronal zigzag incision versus a conventional straight incision pattern for craniotomy. J Neurosurg.

[26] Kłobuszewski B, Szmygin M, Nieoczym K, Kłobuszewska O, Woźniak S, Pyra KK (2024). Advances in treating cesarean scar pregnancy: a comprehensive review of techniques, clinical outcomes, and fertility preservation. Med Sci Monit.

[27] Poelchow F, Codde J, Kendell R, Edgar DW, Wood FM (2024). A randomised investigation of film-forming silicone gel in superficial partial thickness face and neck burn patients: indication of improved early scar pigmentation outcomes. Burns.

[28] Shen Y, He J, Liu JZ (2024). [A randomized controlled trial on the effect of early eschar dermabrasion combined with antimicrobial soft silicone foam dressing in the treatment of deep partial-thickness burn wounds in children]. Zhonghua Shao Shang Yu Chuang Mian Xiu Fu Za Zhi.

[29] Sinha S, Gabriel VA, Arora RK (2024). Interventions for postburn pruritus. Cochrane Database Syst Rev.

[30] McLaughlin J, Branski LK, Norbury WB, Bache SE, Chilton L, El-Muttardi N, Philp B (2018). 60 - Laser for burn scar treatment. Total Burn Care (Fifth Edition).

[31] Klifto KM, Asif M, Hultman CS (2020). Laser management of hypertrophic burn scars: a comprehensive review. Burns Trauma.

[32] Patil UA, Dhami LD (2008). Overview of lasers. Indian J Plast Surg.

[33] Haedersdal M, Moreau KE, Beyer DM, Nymann P, Alsbjørn B (2009). Fractional nonablative 1540 nm laser resurfacing for thermal burn scars: a randomized controlled trial. Lasers Surg Med.

[34] Lin JY, Warger WC, Izikson L, Anderson RR, Tannous Z (2011). A prospective, randomized controlled trial on the efficacy of fractional photothermolysis on scar remodeling. Lasers Surg Med.

[35] Taudorf EH, Danielsen PL, Paulsen IF, Togsverd-Bo K, Dierickx C, Paasch U, Haedersdal M (2015). Non-ablative fractional laser provides long-term improvement of mature burn scars--a randomized controlled trial with histological assessment. Lasers Surg Med.

[36] Weshahy RH, Aly DG, Shalaby S, Mohammed FN, Sayed KS (2020). Clinical and histological assessment of combined fractional CO2 laser and growth factors versus fractional CO2 laser alone in the treatment of facial mature burn scars: a pilot split-face study. Lasers Surg Med.

[37] Xi WJ, Zhang Z, Li J (2021). [Clinical effect of fractional carbon dioxide laser in the treatment of contracture scars]. Zhonghua Shao Shang Za Zhi.

[38] Yang L, Li N, Cheng J, Han JT, Hu DH (2021). [A prospective randomized controlled clinical study on the optimal treatment interval of pulsed dye laser in treating hypertrophic scar after burn]. Zhonghua Shao Shang Za Zhi.

[39] Ge X, Sun Y, Lin J, Zhou F, Yao G, Su X (2022). Effects of multiple modes of UltraPulse fractional CO2 laser treatment on extensive scarring: a retrospective study. Lasers Med Sci.

[40] Alam M, Han S, Pongprutthipan M (2014). Efficacy of a needling device for the treatment of acne scars: a randomized clinical trial. JAMA Dermatol.

[41] Rana S, Mendiratta V, Chander R (2017). Efficacy of microneedling with 70% glycolic acid peel vs microneedling alone in treatment of atrophic acne scars-a randomized controlled trial. J Cosmet Dermatol.

[42] Ali B, ElMahdy N, Elfar NN (2019). Microneedling (Dermapen) and Jessner's solution peeling in treatment of atrophic acne scars: a comparative randomized clinical study. J Cosmet Laser Ther.

[43] An MK, Hong EH, Suh SB, Park EJ, Kim KH (2020). Combination therapy of microneedle fractional radiofrequency and topical poly-lactic acid for acne scars: a randomized controlled split-face study. Dermatol Surg.

[44] Afra TP, Razmi T M, Narang T, Dogra S, Kumar A (2019). Topical tazarotene gel, 0.1%, as a novel treatment approach for atrophic postacne scars: a randomized active-controlled clinical trial. JAMA Facial Plast Surg.

[45] Mujahid N, Shareef F, Maymone MB, Vashi NA (2020). Microneedling as a treatment for acne scarring: a systematic review. Dermatol Surg.

[46] Heitmiller K, Wang JV, Murgia RD, Saedi N (2021). Utility of platelet-rich plasma for treatment of striae distensae: a current exploration. J Cosmet Dermatol.

[47] Suwanchinda A, Nararatwanchai T (2022). The efficacy and safety of the innovative cold atmospheric-pressure plasma technology in the treatment of striae distensae: a randomized controlled trial. J Cosmet Dermatol.

[48] Ibrahim ZA, El-Tatawy RA, El-Samongy MA, Ali DA (2015). Comparison between the efficacy and safety of platelet-rich plasma vs. microdermabrasion in the treatment of striae distensae: clinical and histopathological study. J Cosmet Dermatol.

[49] Ebrahimi Z, Alimohamadi Y, Janani M, Hejazi P, Kamali M, Goodarzi A (2022). Platelet-rich plasma in the treatment of scars, to suggest or not to suggest? A systematic review and meta-analysis. J Tissue Eng Regen Med.

[50] Rasulov MF, Vasilchenkov AV, Onishchenko NA (2005). First experience of the use bone marrow mesenchymal stem cells for the treatment of a patient with deep skin burns. Bull Exp Biol Med.

[51] Roohaninasab M, Seifadini A, Atefi N (2022). Evaluating the effectiveness of stromal-vascular fraction (SVF) cells along with subcision method in the treatment of acne scars: a double-blind randomized controlled clinical trial study. J Cosmet Dermatol.

[52] Xu Y, Huang S, Fu X (2012). Autologous transplantation of bone marrow-derived mesenchymal stem cells: a promising therapeutic strategy for prevention of skin-graft contraction. Clin Exp Dermatol.

[53] Yoshikawa T, Mitsuno H, Nonaka I (2008). Wound therapy by marrow mesenchymal cell transplantation. Plast Reconstr Surg.

[54] Mansilla E, Marín GH, Berges M (2015). Cadaveric bone marrow mesenchymal stem cells: first experience treating a patient with large severe burns. Burns Trauma.

[55] Rasaii S, Sohrabian N, Gianfaldoni S (2019). Intralesional triamcinolone alone or in combination with botulinium toxin A is ineffective for the treatment of formed keloid scar: a double blind controlled pilot study. Dermatol Ther.

[56] Shaarawy E, Hegazy RA, Abdel Hay RM (2015). Intralesional botulinum toxin type A equally effective and better tolerated than intralesional steroid in the treatment of keloids: a randomized controlled trial. J Cosmet Dermatol.

[57] Elhefnawy AM (2016). Assessment of intralesional injection of botulinum toxin type A injection for hypertrophic scars. Indian J Dermatol Venereol Leprol.

[58] Zhibo X, Miaobo Z (2009). Intralesional botulinum toxin type A injection as a new treatment measure for keloids. Plast Reconstr Surg.

[59] Kim SH, Lee SJ, Lee JW, Jeong HS, Suh IS (2019). Clinical trial to evaluate the efficacy of botulinum toxin type A injection for reducing scars in patients with forehead laceration: a double-blinded, randomized controlled study. Medicine (Baltimore).

[60] Ziade M, Domergue S, Batifol D, Jreige R, Sebbane M, Goudot P, Yachouh J (2013). Use of botulinum toxin type A to improve treatment of facial wounds: a prospective randomised study. J Plast Reconstr Aesthet Surg.

[61] Huang RL, Ho CK, Tremp M, Xie Y, Li Q, Zan T (2019). Early postoperative application of botulinum toxin type A prevents hypertrophic scarring after epicanthoplasty: a split-face, double-blind, randomized trial. Plast Reconstr Surg.

[62] Lee SJ, Jeong SY, No YA, Park KY, Kim BJ (2015). Combined treatment with botulinum toxin and 595-nm pulsed dye laser for traumatic scarring. Ann Dermatol.

[63] Chang CS, Wallace CG, Hsiao YC, Chang CJ, Chen PK (2014). Botulinum toxin to improve results in cleft lip repair: a double-blinded, randomized, vehicle-controlled clinical trial. PLoS One.

